# Premedication with Lugol’s solution in total thyroidectomy for graves’ disease and toxic multinodular goiter: is it still indicated?

**DOI:** 10.1007/s13304-025-02475-9

**Published:** 2025-12-19

**Authors:** D. Ciriotto, S. Bernardi, R. Eramo, V. Calabrò, A. Modica, N. de Manzini, C. Dobrinja

**Affiliations:** 1 Division of General Surgery, ASUGI, Cattinara Teaching Hospital, Trieste, Italy; 2 Division of General Surgery, Department of Medicine, Surgery and Health Sciences, Cattinara Teaching Hospital, University of Trieste, Strada di Fiume 447, 34149 Trieste, Italy; 3 SS Endocrinologia, UCO Medicina Clinica, ASUGI, Cattinara Teaching Hospital, Trieste, Italy

**Keywords:** Lugol’s solution, Total thyroidectomy, Graves’ disease, Toxic multinodular goiter, Premedication, Surgical complications.

## Abstract

Premedication with Lugol’s solution (LS) has traditionally been used to reduce the vascularization and friability of the thyroid gland before total thyroidectomy in patients with Graves’ disease (GD) and toxic multinodular goiter (TMNG) with thyrotoxicosis and/or with undetectable serum TSH. However, the effectiveness and applicability of this treatment remain subjects of debate. This study aims to evaluate the surgical and postoperative outcomes in patients premedicated with LS compared to those who were not premedicated. Data from 100 patients who underwent total thyroidectomy for GD and TMNG at our center from 2014 to 2024 were analyzed. Patients were divided into two groups: Lugol+, premedicated with LS (*n* = 57), and Lugol−, not premedicated (*n* = 43). Variables analyzed included thyroid diameter, thyroid weight, operative time, postoperative hemorrhage, hypocalcemia, recurrent laryngeal nerve palsy, length of hospital stay, rate of reintervention for hemorrhage, intraoperative thyroid consistency. No statistically significant differences were found between the groups regarding postoperative hemorrhage (1.7% in Group Lugol+ vs. 2.3% in Group Lugol−), operative time (median: 95 vs. 85 min), immediate postoperative complications such as transient hypoparathyroidism (15.8% vs. 9.3%) and transient recurrent laryngeal nerve (RLN) palsy (3.5% vs. 2.3%), nor the other variables analyzed. Our data suggest that routine preoperative preparation with LS may not be mandatory. This study supports the thesis that patients with GD and TMNG who cannot be premedicated due to inability to obtain LS, insufficient time for preoperative preparation, or lesser compliance by patient, may still be eligible for surgery.

## Introduction

Graves’ disease is an autoimmune disease characterized by the presence of autoantibodies in patients’ serum against the thyrotropin receptor (TRAb), which cause overproduction and release of thyroid hormones. Epidemiologically, it is the most frequent cause of hyperthyroidism in geographical areas of iodine sufficiency; it has a female predominance with a female-male ratio of 4:1, a peak of incidence at 40–69 years [1], and a population prevalence of 1% to 2% [2]. Clinical presentation combines the characteristics of both hyperthyroidism and underlying autoimmunity. The diagnosis is based on characteristic clinical features and biochemical abnormalities that are essentially low or suppressed thyroid-stimulating hormone (TSH) and high free-triiodothyronine (fT3) and free-thyroxine (fT4) levels. The thyroid radioactive uptake test and the dosage of serum TRAb confirm the diagnosis unless it is clinically established. The treatment of Graves’ disease consists of any of three effective and relatively safe possible options: antithyroid drugs (ATDs) such as methimazole, carbimazole or propylthiouracil, radioactive iodine ablation (RAI), and surgery [3]. It is preferred to perform a total thyroidectomy in several clinical situations, such as intolerance, ineffectiveness or recurrence after ATD treatment, contraindication of radioiodine therapy or recurrence after RAI, symptomatic compression or large goiters (≥ 80 g) or coexisting moderate-to-severe active Graves’ orbitopathy, documented or suspected thyroid malignancy. Thyroidectomy is also preferred if one or more thyroid nodules larger than 4 cm are detected or if a thyroid nodule is non-functioning or hyperfunctioning on ^123^I or ^99m^Tc pertechnetate scan. Coexisting hyperparathyroidism requiring surgical treatment or women planning a pregnancy within 6 months are other causes of preference for surgical treatment [4].

Toxic multinodular Goiter (TMNG) is a condition in which multiple nodules in the thyroid gland become overactive, leading to hyperthyroidism, and it is more common in older female adults and in areas with iodine deficiency [5]. The diagnostic criteria are the clinical signs and symptoms of hyperthyroidism (palpitations, weight loss, heat intolerance, tremors, and anxiety), the presence of visibly enlarged thyroid gland with eventually palpable nodules, low or suppressed TSH, and high fT3 and fT4, and the presence of multiple nodules in the thyroid gland at ultrasound (US). The thyroid radioactive uptake scan may help to perform differential diagnosis with GD, and fine-needle aspiration biopsy (FNA) is essential to define the suspicion of malignancy of the detected nodules [5]. The treatment for TNMG typically includes RAI therapy or surgical intervention. ATDs are useful for maintaining lower thyroid hormone levels while preparing for definitive treatment. Surgery is preferred in cases where goiter compression causes symptoms, suspicion of malignancy exists, or RAI therapy is contraindicated [6].

If surgery is selected as treatment, both in the case of underlying GD or TMNG, it is important to perform the intervention in a long-experienced high-volume thyroid surgery center, and careful preoperative management is crucial to optimize surgical outcomes. Pre-treatment with ATDs is strongly recommended by international guidelines [6, 7] to achieve the euthyroid state in order to avoid the risk of precipitating thyroid storms during surgery. Euthyroid state is generally achieved after a few weeks of ATD treatment. Beta-blockers are often added effectively to control tachycardia and palpitations as hyperthyroid symptoms. Premedication with Lugol’s solution (LS) has been a standard practice to minimize vascularity and fragility of the thyroid gland in patients with GD and TMNG undergoing total thyroidectomy.

LS was developed in 1829 by the French physician Jean Guillaume August Lugol. It was initially used as a cure for tuberculosis. It is a solution of elemental iodine (5%) and potassium iodide (KI, 10%) mixed with distilled water. It had many uses, such as in histologic preparations, in the diagnosis of cervical cell alterations, in Schiller’s test, or in dental procedures. Already in the 1920s literature, there is evidence that LS was given as a pre-treatment for thyroid surgery [8], so its use as premedication became routine. Due to the high concentration of iodine, LS inhibits thyroxine and tri-iodothyronine synthesis and thyroid secretion, the former by blocking thyroperoxidase by the Wolff–Chaikoff effect. Furthermore, these effects are obtained with rapid onset [6, 9].

The preoperative preparation of patients with GD with iodine-rich preparations was proposed by Plummer in 1923 in order to reduce intraoperative bleeding and prevent the perioperative “thyroid storm” due to the massive release of thyroid hormones during surgery [10, 11], observing a 75% decrease in thyroidectomy-associated mortality at that time [8]. Already in 1925, intrathyroid blood vessel compression was described after LS therapy [12] as a complement to the effect of fT4 and fT3 reduction. The reduction of blood loss is associated with a 60% reduction in systemic angiogenic factor and with 50% reduction of interleukin-16 [11]. Therefore, the rationale behind LS use in preoperative preparation of patients undergoing total thyroidectomy for GD and TMNG is to reduce intraoperative blood loss and facilitate surgical manipulation. Despite its long-standing use, recent advances in surgical techniques and preoperative management have called into question the necessity of LS in all cases [13, 14].

This study aims to critically evaluate the impact of Lugol’s solution on surgical and postoperative outcomes in total thyroidectomy for GD and TMNG with thyrotoxicosis and suppressed TSH.

## Materials and methods

A retrospective cohort study was conducted on 100 consecutive patients who underwent total thyroidectomy for GD and TMNG at our institution from 2014 to 2024. The patients were divided into two groups based on preoperative preparation: Lugol+: 57 patients premedicated with Lugol’s solution, 42 women (74%) and 15 men (26%), mean age 58 years. Lugol−: 43 patients who did not receive premedication with Lugol’s solution, 34 women (79%) and 9 men (21%), mean age 52 years.

Selection criteria for Lugol+ or Lugol – were: patients’ compliance/preference, availability of Lugol Solution at time of intervention (in preoperative period), and adequate time for Lugol administration.

The surgical team was the same for every patient and the surgical technique was constant during the period of the study. In our centre, LS was routinely administered as preoperative premedication until December 2019. The regimen consisted of 7 drops, taken three times daily for 10 days prior to surgery. Due to a radical change in the organization of surgical sessions starting in January 2020, a complete premedication regimen has been unfeasible since that time. We were confident in omitting LS premedication thanks to the statistically significant data demonstrated by the modern literature [13, 15, 16].

Data were collected on age, gender, preoperative thyroid hormones dosage (TSH, fT3, fT4), also expressed in a number of patients in euthyroid or hyperthyroid status at the preoperative time, preoperative thyroid dimension (based on the craniocaudal and anteroposterior diameter of the greater thyroid lobe at the preoperative US or CT-scan), FNA with cytological examination following SIAPEC 2014 classification [17] in case of concomitant presence of thyroid nodules, consistency of the thyroid tissue found at surgical intervention, operative time, postoperative haemorrhage or hematoma requiring reintervention for a haemostatic procedure, hypoparathyroidism (transient or permanent), recurrent laryngeal nerve (RLN) palsy (transient or permanent), drainage amount on the first postoperative day (1st POD), the permanence of drainage, length of hospital stay, diameter and weight of thyroid gland at the pathologist’s examination. The hyperthyroid state was defined by fT4 level over 12.0 pg/mL with low (< 0.40 µUI/mL) or suppressed TSH. Non-hyperthyroid patients were classified as euthyroid. Hypocalcemia was defined as serum calcium < 8.0 mg/dl. We defined permanent hypoparathyroidism as low parathyroid hormone (PTH) levels (< 11 pg/mL according to our laboratory’s cut-off, that uses a third-generation method) with hypocalcemia lasting beyond six months post-surgery. Haemorrhage was defined as a postoperative complication when the patient required haemostatic procedures (reintervention). Haematoma was defined as a postoperative lesser bleeding with no need for reintervention.

Preoperative imaging (ultrasound) was consistently conducted by the same radiologist, a member of the multidisciplinary team specializing in thyroid diseases, who reported the dimensions of the thyroid lobes in accordance with our internal standard protocols. Each patient had almost two preoperative ultrasounds: one performed before our visit and another performed preoperatively, by our dedicated endocrine radiology (always the same operator). The time interval between the ultrasound and surgery was about 45 days.

To determine thyroid gland size in pre- and postoperative period, we preoperatively measured in millimeters (mm) the thyroids’ diameter lobe at US and we measured in mm the thyroid’s diameter lobe at histology. In the histological report, there always reported also the thyroid weight that may offer another additional information.

All patients underwent laryngoscopy before and after all surgical procedures to evaluate vocal cord motility. Permanent RLN palsy was defined as the persistence of chordal hypomotility or non-motility at six months of follow-up. In case of recurrent laryngeal nerve palsy, early voice therapy was started with treatment with corticosteroid and vitamin B complex. The laryngeal nerve intraoperative monitoring was performed in all operations [18]. We used the Intermittent Intraoperative Neuromonitoring [I-IONM]. Advanced bipolar coagulation devices were routinely utilized in every surgical procedure.

In order to assess the effect of LS on the consistency and the dimension of the thyroid, we compared the consistency of thyroid tissue and the variation between the preoperative and the postoperative dimension of the gland.

To assess the consistency of thyroid tissue encountered during surgical procedures (classified as abnormal versus normal), we retrospectively reviewed all operative reports. We specifically selected cases involving “difficult thyroidectomies,” defined as procedures in which surgeon documented that the thyroid gland was either increased in consistence, with adherence to adjacent structures such as the trachea and/or prethyroid muscles, or excessively friable, with hypervascularization of the gland. These cases were classified as having “abnormal” thyroid tissue.

We reviewed all surgical reports and also all preoperative ultrasonography to check if information’s regarding hypervascularization or echogenicity in thyroid parenchyma. In addition, in the histological reports we checked the macroscopic description of thyroid tissue to determine better the thyroid consistence at time of operation”. The number of cases exhibiting increased tissue and gland friability were also compared separately.

### Follow-up

At least a 6 months follow-up was achieved for all patients. In the case of post-operative serum calcium levels lower than 8.0 mg/dl, patients were treated with substitutive therapy with Calcium carbonate tablets (1–3 tablets/day) and with calcitriol (1-alpha, 25-dihydroxy vitamin D3) 0.5 mg × 2/day. In case of symptomatic hypoparathyroidism, the serum calcium levels were adjusted with intravenous administration calcium gluconate.

Serum calcium levels were re-evaluated every three days following hospital discharge in hypocalcemic patients, until normalization of serum calcium and PTH levels was achieved. The follow-up ended after the normalization of PTH and calcium levels.

All patients with transient RLN palsy were checked with laryngoscopy after voice therapy.

### Ethical aspects

All data were anonymously collected in a protected electronic database. The study has been conducted according to Good Clinical Practice, to ethical principles from the Helsinki Declaration. The informed consent was signed by every patient enrolled. The study has been registered by the Local Ethical Committee with the following protocol number 431_2025H.

### Statistical analysis

The statistical analysis was performed by using the following tests: the Shapiro-Wilk normality test to verify if the numerical data followed the normal distribution or not, the t-student test for normal-distributed variables (age and preoperative thyroid dimension), the Mann-Whitney test for the not-normal-distributed variables (TSH, fT3, fT4, operative time, length of hospital stay, thyroid’s weight, postoperative thyroid dimension); the Chi-square test for gender, rate of overall postoperative complications, preoperative euthyroid state and consistency of thyroid tissue and the Fisher exact test for the rate of hypoparathyroidism, postoperative haemorrhage, and recurrent laryngeal nerve palsy; the T-Test for paired data to assess the significance of the variation between preoperative and postoperative dimensions of the gland. The software used was RStudio. A p-value less than 0.05 was considered statistically significant.

## Results

We enrolled 100 patients with a mean age of 54 years old and male to female ratio of 1:3.1. The two groups (Lugol+; Lugol−) were homogeneous with respect to preoperative diagnosis (GD and TMNG), thyroid weight, and dimension both at preoperative US or CT-scan and at the mesurement performed by the Pathologist postoperatively, with median weight 43 g in Lugol+ group vs. 46.5 g Lugol− group and mean greater diameter 78,5 mm vs. 69,1 mm at preoperative US or CT scan and median greater diameter 63 mm vs. 62,5 mm at postoperative pathological examination.

The comparison of outcomes between the two groups, Lugol+ and Lugol− showed no statistically significant differences in the following parameters: preoperative TSH, fT3 and fT4 dosage, rate of patients in preoperative euthyroid state (Table 1), operative time, drainage amount on 1st POD, permanence of drainage and length of hospital stay. Postoperative haemorrhage or hematoma and reintervention rate did not show any statistically significant differences (all patients with postoperative haemorrhage underwent surgery for haemostasis or evacuation. No haematoma was reported). Also, transient hypoparathyroidism rate, transient recurrent laryngeal nerve palsy rate, other complications rate such as surgical site infection or wound dehiscence, and overall complications rate were comparable between the two groups (Table 2). On the short-term follow-up for RLN injury and hypoparathyroidism, no cases of permanent recurrent laryngeal nerve palsy were detected. There were 2 cases (1 patient for each group) of definitive (> 6 months) hypoparathyroidisms. There wasn’t any recurrence in our follow-up period (Table 1).

**Table 1:** Demographic and preoperative characteristics of the study population

		Lugol+ (57pts) 34 GD (59.6%) 23 TMNG (40.4%)	Lugol− (43 pts) 33 GD (76.7%) 10 TMNG (23.3%)	*p*-value 0.11
Gender (female/male)		42/15 (2.8:1)	34/9 (3.8:1)	0.70
Age (years), ma (range)		58 (42–75)	52 (36–69)	0.09
Thyroid preop. Dim. (mm), ma (range)		77.0 (56–91)	69.1 (50–89)	0.38
Thyroid postop. Dim. (mm), me (IQR)		63 (50–85)	65 (51–75)	0.49
Gland’s consistency: GD only abnormal/normal n/n (%/%) GD + TNMG		19/15 (55.9/44.1%) 19/38 (33.3/66.7%)	13/20 39.4/60.6%) 14/29 (32.6/67.4%)	0.29 1
Gland’s consistency (GD only)	Increased consistency, with adherence yes/no (%/%)	16/18 (47.0/53.0%)	8/25 (24.2/75.8%)	0.07
	Friable, hypervascular yes/no (%/%)	4/30 (11.7/88.3%)	5/28 (15.1/84.8%)	0.73
Thyroid weight (g), me (IQR)		42 (26–117.5)	46.2 (27–63)	0.59
TSH (µUI/mL), me (IQR)		0.88 (< 0.01–2.65)	0.83 (< 0.01–2.73)	0.90
fT3 (pg/mL), me (IQR)		3.6 (3.2–4.2)	3.5 (3.1–4.7)	0.73
fT4 (pg/mL), me (IQR)		7.2 (6.3–10.4)	7.95 (7.0–9.2)	0.78
Preop. Euthyroidism (n% - n/N)		84%−48/57	88%−38/43	0.67

Preop = preoperative; dim = dimension; ma = mean; me = median; TSH = thyroid-stimulating hormone: normal range 0.40-4.00 μUI/mL; fT3 = free tri-iodothyronine: normal range 2.0-3.5 pg/mL; fT4 = free tyroxine: normal range 5.6-12.0 pg/mL; IQR = Interquantile range; n% = percentage; n/N = number of cases/totality of cases

**Table 2:** Surgical outcomes in Lugol+ and Lugol– groups

	Lugol+ (57pts) 34 GD (59.6%) 23 TMNG (40.4%)	Lugol− (43 pts) 33 GD (76.7%) 10 TMNG (23.3%)	*p*-value 0.11
Haemorrhage/Haematoma (n% - n/N)	1.7%–1/57*	2.3%–1/43	1
Tr. Hypoparathyroidism (n% - n/N)	15.8%–9/57	9.3%–4/43	0.38
Transient RLN palsy (n% - n/N)	3.5% –2/57	2.3%–1/43	0.79
Others (n% - n/N)	3.5%–2/57	4.7%–2/43	1
Overall complications (n% - n/N)	24.5%–14/57	18.6%–8/43	0.22
Operative Time (min.), me (IQR)	95 (75–115)	85 (67–100)	0.11
Drain on 1st POD (mL), me (IQR)	40 (20–70)	30 (30–50)	0.31
Perm. of Drain (days), me (IQR)	2 (2–3)	3 (2–3)	0.09
Length of HS (days), me (IQR)	3 (2–4)	3 (2–3)	0.74

*Requiring surgical evacuation; Min.=minutes; n%=percentage; n/N = number of cases/total of cases; tr = transient; RLN = recurrent laryngeal nerve; me = median; drain = drainage amount; 1st POD = first postoperative day; perm = permanence; HS = hospital stay; IQR = Interquantile range

In particular, regarding the postoperative morbidity, were found between the groups regarding postoperative haemorrhage (1.7% in Group Lugol+ vs. 2.3% in Group Lugol−), operative time (median: 95 in Lugol+ vs. 85 min in Lugol−), immediate postoperative complications such as transient hypoparathyroidism (15.8% in Group Lugol+ vs. 9.3% in Group Lugol−) and transient recurrent laryngeal nerve (RLN) palsy (3.5% in Group Lugol+ vs. 2.3% in Group Lugol−). Length of hospitalization was mean 2.82 days (range: 1–8) for the group Lugol+ and 3.025 days (range: 2–6 days) for the group Lugol−.

The proportions of GD and TMNG were 57.9% (33 patients) and 42.1% (31 patients) in the Lugol+ group, whereas they were 72.1% (24 patients) and 27.9% (12 patients) in the Lugol – group. In the Lugol+ group, 5 patients with GD also had single thyroid nodules detected (2 TIR3A, 1 TIR3B, 1 TIR4, and 1 TIR5 on preoperative FNA). Only one of these (TIR5) was confirmed as papillary carcinoma on definitive histology; the others were benign. In 4 cases, an occult papillary carcinoma with a major diameter of less than 1 cm was diagnosed on histology. Three of these were concomitant with GD, and one with TMNG. In the Lugol– group, 4 patients with GD also had single thyroid nodules detected (1 TIR3B, 3 TIR4). Two of these (both TIR4) were confirmed as papillary carcinomas on histology, while the others were benign. In 3 cases, an occult papillary carcinoma with a major diameter of less than 1 cm was diagnosed on histology; 2 were concomitant with GD and one with TMNG (Table 2).

The review of the operative reports allowed for the identification of the rate of “difficult thyroidectomies,” defined as cases in which the operating surgeon reported that the thyroid was either increased in consistency or adherent to surrounding tissues, excessively friable with hypervascularisation. In the Lugol+ group, 19 reports (33.3%) documented abnormal thyroid tissue, while 38 reports (67.7%) described normal tissue. Conversely, in the Lugol − group, 14 (32.6%) reports indicated abnormal tissue, and 29 reports (67.4%) described normal tissue. When stratifying the patients into GD and TMNG groups, we observed that among TMNG patients, no abnormal thyroid tissue was reported in the Lugol+ subgroup (0/23, 0%), whereas one abnormal case was reported in the Lugol − subgroup (1/10, 10%). For GD patients, 19 operative reports in the Lugol+ group described abnormal tissue and 15 described normal tissue (55.9% abnormal vs. 44.1% normal), compared to 13 abnormal and 20 normal reports in the Lugol − group (39.4% abnormal vs. 60.6% normal). The difference between these groups was not statistically significant (*p* = 0.29).

Considering only patients diagnosed with Graves’ disease, we compared the frequency of reported increased tissue consistency with adherence. In the Lugol+ subgroup, 16 cases reported this condition, while 18 did not. Conversely, in the Lugol− subgroup, 8 cases reported increased tissue consistency, and 25 did not, with no statistically significant difference (*p* = 0.07). Regarding increased friability and hypervascularization, 4 cases in the Lugol+ subgroup reported these features, compared to 30 who did not; in the Lugol− subgroup, 5 cases reported these features, while 28 did not, without any statistically significant difference (*p* = 0.73).

The comparison between preoperative and postoperative thyroid gland dimensions revealed a statistically significant reduction only in the Lugol− group (mean diameter reduction of 4.15 mm; *p* = 0.003). In contrast, the Lugol+ group showed a non-significant reduction (mean diameter reduction of 3.33 mm; *p* = 0.062). When considering only patients with GD, both groups exhibited non-significant reductions in gland size (Lugol –: 2.14 mm; *p* = 0.16; Lugol+: 1.67 mm; *p* = 0.37).

## Discussion

This study aimed to evaluate the impact of omitting preoperative LS in the surgical management of thyrotoxic patients undergoing total thyroidectomy over a ten-year period (2014–2024). The role of ATDs as a first-line treatment for TMNG and GD remains a subject of debate. While some consider ATDs as definitive therapy, others view them as second-line options, especially given the variable nodule sizes and iodine uptake that characterize these conditions. Typically, total thyroidectomy is performed for GD and TMNG, often after achieving a euthyroid state through ATD therapy, using agents such as methimazole, carbimazole, or propylthiouracil. In our series, however, surgical intervention was sometimes undertaken despite persistent thyrotoxicosis, due to inadequate hormonal control, and in cases of severe ophthalmopathy associated with Basedow’s disease or non-responsive TMNG with suppressed TSH levels. Preoperative assessment included cervical imaging, such as US or CT scan, the latter in patients with tracheal deviation or mediastinal extension.

Although existing literature suggests that LS administration reduces perioperative bleeding by decreasing gland vascularity [19], our findings did not support this benefit. The primary surgical expectation from LS use is to facilitate dissection by reducing gland vascularity and friability, thereby making identification and preservation of the RLN and parathyroid glands easier. Nevertheless, our data indicate a trend toward higher incidences of hypoparathyroidism and RLN palsy in the LS-treated group, although these differences did not reach statistical significance. This discrepancy may be attributable to the limited sample size, which reduces statistical power, and suggests that a larger cohort could reveal significant differences. The retrospective design also imposed limitations, notably the inability to quantify changes in thyroid gland size and vascularity pre- and post-LS administration “in vivo”, as only a single preoperative ultrasound or CT was available. Postoperative pathology reports provided the sole macroscopic measure of gland size, revealing no statistically significant reduction following LS, and a mean decrease of only 4.15 mm in the subgroup not receiving LS. Therefore, we cannot conclude that patients who underwent surgery without Lugol’s solution preparation, yet continued their ATDs prescribed at the time of diagnosis, would derive a clinically meaningful benefit compared to those prepared with Lugol’s iodine in addition. The retrospective design of this study may have limited the statistical significance of these findings, partly due to the extent of missing data. A larger dataset could potentially enhance the robustness and significance of the results. Additionally, gland size may vary between pre- and postoperative measurements due to complete devascularization after surgery, independently of Lugol’s solution use.

Our findings align with recent randomized trials and meta-analyses [13–16], which have reported that preoperative LS does not significantly influence intraoperative blood loss, operative time, or postoperative complications such as RLN palsy and hypoparathyroidism, despite effectively lowering thyroid hormone levels. For instance, Schiavone et al. [14] demonstrated that LS reduces T3 and T4 concentrations but does not impact vascularity or surgical morbidity. Variability in treatment protocols, including drug dosing and timing of ATD therapy, likely contributes to the lack of consensus regarding LS use. Many studies rely on indirect or subjective assessments, further complicating definitive conclusions [14, 20, 21]. 

Given these observations, routine preoperative LS administration may not be necessary in the modern surgical setting. Our results suggest that meticulous surgical technique and careful perioperative management can sufficiently mitigate the risks traditionally associated with hypervascular thyroid glands, rendering LS supplementation optional. This approach could be particularly advantageous in scenarios where LS availability is limited, preoperative preparation time is constrained, or patient compliance is questionable.

It is important to acknowledge potential confounders inherent in our study. The extended timeframe encompasses significant evolutions in surgical practice, notably the widespread adoption of energy-based vessel sealing devices, which have improved haemostasis and may have influenced outcomes independently of LS use. In the latter years of the study, these devices likely contributed to reduce bleeding and operative time, potentially obscuring the effects attributable solely to LS omission. Nevertheless, the surgical team maintained consistent techniques throughout, and intraoperative detection of parathyroid glands was not augmented by advanced imaging or fluorescence-guided methods.

The assessment of thyroid tissue consistency during surgery is inherently subjective and difficult to standardize. In this study, we hypothesized, based on existing literature, that LS may reduce vascularization and enhance the consistency of thyroid tissue, which tends to become more friable due to GD. Consequently, we expected a lower prevalence of friable tissue and thyroid hypervascularization intraoperatively in the LS-treated group. However, our findings do not support this hypothesis. Increased tissue consistency due to fibrotic sclerosis can be encountered during thyroidectomy procedures, and there is no evidence to suggest that LS confers any benefit in such cases. However, this intraoperative finding must be considered a potential confounder, as it may cause prolongation of operative time, and potentially increased risk of complications. It is important to note that various other factors can influence thyroid tissue consistency—such as underlying thyroiditis, malignancy, prior inflammation, exposure to ionizing radiation, and patient habitus—regardless of Lugol’s use. Consequently, drawing definitive conclusions regarding the true effects of LS on tissue consistency presents significant challenges. Implementing a more standardized and detailed classification of intraoperative difficulties, along with the systematic application of objective assessment tools (such as elastography or histological scoring), would enhance the robustness of the analysis and facilitate improved comparisons in future prospective studies.”

Another limitation of the study could be that we did not randomize the patients, but in our setting (general surgery institute) it is not always possible to inform patients in advance of the surgery date, and therefore Lugol’s solution was administered to patients who were compliant and for whom there was enough time to administer it.

One more aspect warrants consideration: patients with GD are often more prone to bleeding tendencies, and the proportion of TMNG patients was substantial (57.9%) in the LS group, reflecting regional variations and clinical practice patterns. Some centers avoid LS in TMNG due to concerns over transient hyperthyroidism exacerbation [22]; however, in our series, surgeries were performed even in persistent hyperthyroidism cases following prolonged ATD therapy.

International guidelines advise achieving a euthyroid state before surgery to prevent intraoperative thyroid storm, and current trends encourage to reduce preoperative pharmacologic preparations in favour of optimized medical control with beta-blockers and ATDs [19]. The prevailing trend in evidence-based medicine appears to be moving even “toward no preparation at all”, with the exception of beta-blocker administration for heart rate control [23]. Ideally, a comparative analysis between prepared and non-prepared patients would provide more definitive insights, with omission of LS representing a subgroup within the latter category. However, in our practice, the Endocrinology team prescribes ATDs as the first-line treatment, and there is no consensus among colleagues regarding the discontinuation of ATDs when surgical intervention is indicated.

Due to the retrospective design of this study, we were unable to directly assess patient adherence to premedication with LS, which may introduce a potential bias. The primary objective of this research was to critically evaluate the impact of LS premedication on surgical and postoperative outcomes in patients undergoing total thyroidectomy for GD and TMNG in real-world clinical settings. Despite our concerted efforts to monitor compliance, absolute certainty regarding adherence to the preoperative preparation regimen remains unattainable within routine practice. Noncompliance with LS premedication has been recognized as a significant challenge [14], often due to patient-reported adverse effects such as a bitter or metallic taste, gastrointestinal symptoms including nausea and vomiting, or, in rare instances, cutaneous reactions reactions, fatigue, or fever [1]. Additionally, LS is predominantly available as a compounded (galenic) formulation rather than a commercially manufactured product, which poses logistical challenges. Many pharmacies may lack the necessary facilities or expertise to prepare such solutions on demand, thereby limiting its widespread routine use. These factors collectively may influence the generalizability of our findings and underscore the need for strategies to improve patient adherence and facilitate the availability of premedication formulations in clinical practice.

## Conclusions

Our findings support the notion that routine preoperative Lugol’s solution may not confer significant advantages in terms of operative ease or complication rates in total thyroidectomy for Graves’ disease and toxic multinodular goiter. Modern surgical techniques and perioperative care appear sufficient to address the challenges posed by hypervascular glands, making Lugol’s solution supplementation optional in selected patients. Routine use of Lugol’s solution may, therefore, be reconsidered, allowing patients who cannot undergo this premedication to be eligible for surgery without increased risk of complications. Future prospective studies with standardized protocols, larger sample sizes, and objective measures of gland vascularity are needed to definitively establish the role of LS in this context.

## Data Availability

Data are available under specific request.
